# An Empirical Exploration of Sports Sponsorship: Activation of Experiential Marketing, Sponsorship Satisfaction, Brand Equity, and Purchase Intention

**DOI:** 10.3389/fpsyg.2021.677137

**Published:** 2021-06-24

**Authors:** Chun-Hua Hsiao, Kai-Yu Tang, Yu-Sheng Su

**Affiliations:** ^1^Department of Marketing, Kainan University, Taoyuan City, Taiwan; ^2^Department of International Business, Ming Chuan University, Taipei, Taiwan; ^3^Department of Computer Science and Engineering, National Taiwan Ocean University, Keelung City, Taiwan

**Keywords:** activation of experiential marketing, sports sponsorships, brand equity, purchase intention, sponsorship satisfaction

## Abstract

The purpose of this study is to investigate the relationship between the activation of experiential marketing, satisfaction with sponsored sporting events, brand equity, and subsequent product purchase intentions in a small-scale sponsorship campaign. Survey data were collected from 238 actual runners in the terminal rest area after they had completed a race. Structural equation modeling with the bootstrap method was carried out to examine the proposed hypotheses. Results revealed that in terms of product purchase intention, brand equity was the most influential factor, followed by experiential marketing activation and satisfaction with the sponsorship. Specifically, experiential marketing activation significantly influenced the sports sponsorship and the sponsor's brand equity; however, satisfaction showed an insignificant effect on purchase intention. In addition, the mediation test shows that brand equity is an important mediator of experience marketing and satisfaction to product purchase intention. Findings provide some empirical insights into how small-scale sponsorship can benefit sponsoring companies, including intangible brand assets and tangible product consumption. The results could encourage more companies to organize small-scale sponsorship races and to present brand-related experiences (e.g., experiencing product packaging, on-the-spot experience areas). In this way, opportunities may be provided to attract more runners (or potential consumers) to participate in the event and have a deeper brand experience. This study contributes to a better understanding of the effectiveness of small-scale sponsorship in Asia to increase the generalization of the small sponsorship literature. Small-scale sporting events can bring public attention and economic benefits to the host company, and encourage more people to participate, thereby resulting in long-term social and health benefits in the community.

## Introduction

After the successful campaign, “Adidas Streetball Challenge” (a series of street basketball tournaments) in 1992, event marketing has successfully attracted the attention of marketing professionals and academics (Nufer, 2015). The terms “event marketing” and “event sponsorship” are often used interchangeably to explain the same phenomenon whereby consumers actively participate in sponsored events (Cornwell, 1995). The global brand spending on sports sponsorships was $46 billion in 2019 (Two Circles, 2019). As we look at global sponsorship spending across regions, the Asia Pacific region was estimated to grow 5.7% ($16.6 billion) in 2018, surpassing the projected 5.1% increase in Europe ($17.6 billion) and other regions. This shows that sponsorship is continuing to grow throughout the world, especially in Asia. However, the amount of worldwide sports sponsorship spending dropped to $29 billion in 2020 due to the new coronavirus epidemic (Two Circles, 2020).

Among different types of sponsorship, sports sponsorship is seen as a powerful tool for brand communication. Shank and Lyberger (2014) considered sports sponsorship as an investment in a sports entity (e.g., athlete, league, or event) to achieve a company's marketing goals or promotional strategies. A large body of literature supports the arguments that sports sponsorship can improve corporate image and enhance consumers' purchase intention regarding the sponsoring brand (e.g., Liu et al., 2015; Eddy and Cork, 2019). In addition, researchers have found that the longer the sponsorship lasts, the stronger the link between the sponsor's brand image and the sponsored sports event in a consumer's memory (Cornwell and Humphreys, 2013).

Most existing literature on sports sponsorship focuses on the positive benefits of large-scale sporting events (e.g., FIFA World Cup, international marathons). It has been confirmed that consumer involvement and experiences of sports sponsorship events can increase public awareness (Jin et al., 2013; Jeong and Kim, 2019), enhance the sponsoring company's brand equity (Zarantonello and Schmitt, 2013), and encourage consumers' future consumption (Addis and Holbrook, 2001). However, Mount and Niro (1995) pointed out that small-scale sponsorship often emphasizes its impact on consumer feedback and the success of the sponsored events to measure the effectiveness of the sponsorship. We believe that small-scale sponsorship can encourage people, and especially amateurs, to participate in sporting events in regional or local communities, including many first-time participants.

In summary, most sponsorship studies have focused on large-scale or mega-sporting events which are sponsored primarily by global brands, such as the attitude toward large-scale sports events and sponsor's brand equity (Lee et al., 2015). Only a few studies have discussed the benefits of small-scale sporting events, for example, small-scale amateur sporting events (Low and Pyun, 2016) and small-scale marathon event participants (Koo et al., 2014). Therefore, it is important to further explore the effectiveness of small-scale sports events, especially in Asia where sports events are in the budding stage, such as in Taiwan. In addition, previous research mainly focused on how consumers' or spectators' attitudes toward events can be transferred to the sponsoring brand. However, little research has examined the effectiveness of event sponsorship from the perspectives of participants. For example, Hickman (2020) found that compared with TV viewers, participants in the sponsor's brand experience demonstrated higher brand awareness. As such, to fill this research gap in the literature, this study focused on actual participants in sports events as the research object. A research framework from the perspective of experiential marketing was proposed for this study to explore the effectiveness of event sponsorship, including sponsorship satisfaction, brand equity, and purchase intention of sponsored products. As such, the present study focuses on a small-scale sponsorship campaign entitled “Shimen Reservoir International Road Run Challenge in Taiwan” to provide empirical evidence of the effectiveness of the small-scale events for the sponsorship literature. The campaign was sponsored by GoHiking, an outdoor products company in Taiwan, for the purpose of promoting environmental protection as well as the corporate brand. Three main purposes of the research are raised as follows: (1) drawing from the experiential marketing perspective to determine whether sponsoring a small-scale sporting event can create strong brand equity for the sponsoring company; (2) drawing from the event marketing perspective to determine whether participants' satisfaction with the sporting event will increase their perceived brand equity of the sponsoring company; and (3) taking both together to determine whether participants' purchase intention regarding the sponsoring company's products will be affected by the above factors.

## Literature Review

### Research on Sport Sponsorship

Meenaghan (1983) defined sponsorship as the provision of financial assistance to an activity by a company in return for exploitable commercial objectives. Mullin et al. (2000) provided a similar definition as the acquisition of rights to directly associate with a product or event to obtain benefits related to that association. During the past decades, sponsorship has been used as a critical marketing tool and has become a major global industry (Alonso-Dos-Santos et al., 2016).

Researchers have reported that some companies which have invested in sports sponsorship received significant growth in their revenue (Blake et al., 2019). This may be attributed to the enhancement of brand visibility and product consumption among sports participants (Kelly et al., 2017). More recently, Kwon and Cornwell (2020) confirmed that sponsoring sports events can enhance the value of sponsoring firms. From the perspective of consumers, participating in sports events can increase their brand engagement and brand experiences, which will evoke their positive attitude and enhance their brand awareness of the sponsoring company (Wang and Kaplanidou, 2013).

Empirical research on large sports events has combined some variables to frame the effects of the sponsorship. For example, from the perspective of the brand's reputation in large-scale companies, Close et al. (2006) proposed that consumers' knowledge about a sponsor's product and community involvement positively affects their opinion of the sponsor's brand. Likewise, Lacey et al. (2010) also stated that the role of consumers' knowledge of the event sponsor's products would enhance consumers' commitment to the sponsor and intentions to purchase the sponsor's products. In contrast, Lee et al. (2015) emphasized the mediating role of emotion (e.g., pleasure, arousal) in the relationship between the sponsor's event attitude and brand equity. Recently, Quintal et al. (2020) explored and compared the impact of sponsorship and non-sponsorship activation of global brands (e.g., Adidas and Nike) on cognitive, affective, and conative behaviors across countries. They found significant differences in the attitude-purchase intentions of participants from different countries.

Researchers have paid great attention to large-scale sports events; however, research on small-scale sports sponsorship is relatively limited. Tzetzis et al. (2014) addressed the quality of the sponsored services (such as access, venue quality, and contest quality) contributing to participant satisfaction. More specifically, Koo et al. (2014) proposed a model of event image-satisfaction-behavioral intentions to capture the effect of small-scale sports sponsorship. They concluded that participants' intention to revisit and recommend the event depended on their perceptions of the event image and their satisfaction with the event. In addition to the previous research focusing on the events themselves (e.g., event quality or event image), Low and Pyun (2016) emphasized the importance of the sponsor's characteristics (e.g., sincerity, credibility), and believed that the outstanding characteristics can enhance a positive attitude toward sponsorship activities, such as sports sponsorship of local and amateur colleges (Ko et al., 2017; Su and Chen, 2020; Su and Lai, 2021; Su and Wu, 2021).

**Figure 1:**
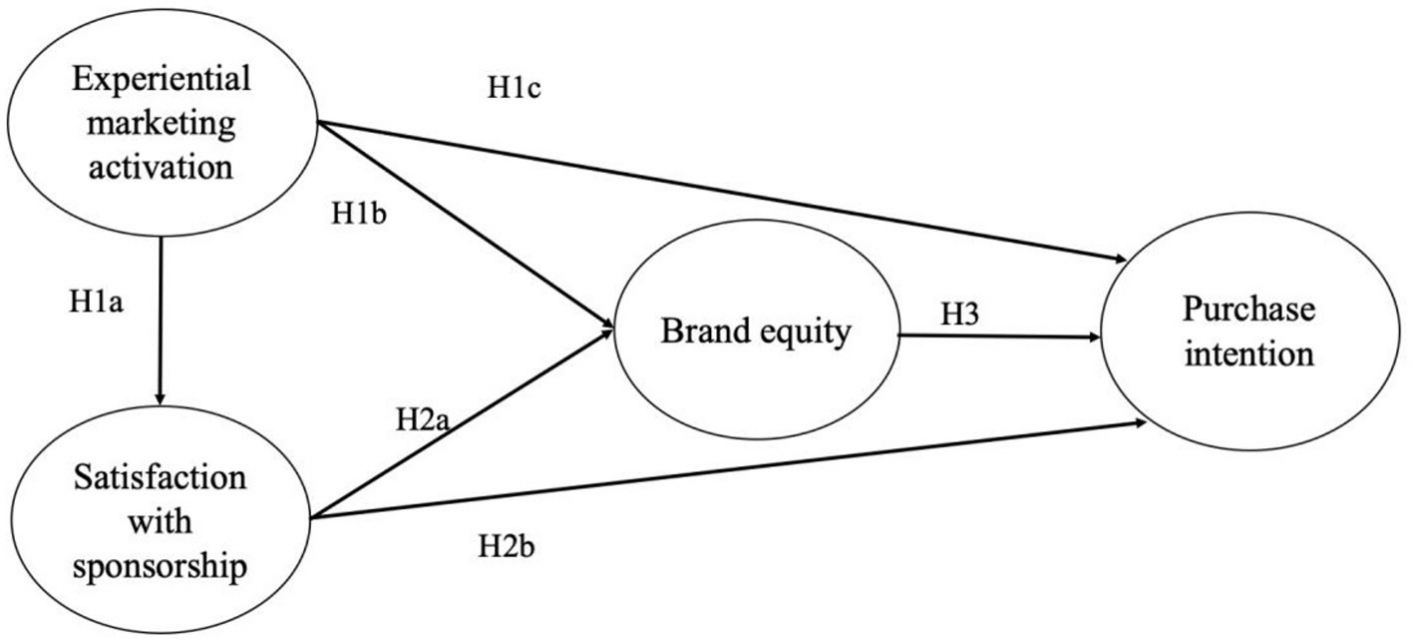
Proposed research framework.

In sum, in large-scale sponsored sports events, consumers' previous knowledge of sponsored products (mainly internationally renowned brands) is critical to brand equity and purchase intentions. On the other hand, in a small sponsorship event, the quality of the event itself is very important for enhancing participants' satisfaction. Cornwell's (2019) research has suggested that the results of consumer-oriented, sponsorship-linked marketing as cognition (e.g., awareness and image), affection (e.g., liking and preference), and behavior (e.g., purchase intention and making purchases). Therefore, participants' engagement and satisfaction have been considered as two common factors that are critical to the effectiveness of sponsorship and behavioral intention (Eagleman and Krohn, 2012). Furthermore, due to the lack of prior knowledge of sponsored products (usually small sponsoring companies), experiential marketing activation strategies are necessary and are believed to have a positive impact on brand equity (Ross et al., 2008). Therefore, based on the previous research on experiential marketing, satisfaction, brand equity, and purchase intention, a research framework for small sports sponsorship was proposed. As shown in Figure 1, we assume that consumers' positive experience of the sponsor's product will have a positive impact on their satisfaction with the sponsorship event. In addition, consumers' experiential marketing activation and sponsorship satisfaction will have a positive impact on the sponsor's brand equity, thereby jointly increasing the willingness to purchase the sponsor's products. This present study aims to contribute to the small sponsorship literature, and the proposed model provides a platform for future study on this emerging small-scale event sponsorship area. Following previous literature, the proposed model is parsimonious (Hair et al., 1998) and focused on the effects of three influential constructs on purchase intention related to small-sponsoring products (Eagleman and Krohn, 2012; Koo et al., 2014; Ko et al., 2017).

### Activation of Experiential Marketing

Customer experiences are critical to the activation of experiential marketing. Meenaghan and O'Sullivan (2013) pointed out that in order to effectively evaluate sponsors, sponsors must provide brand experience, engagement, and involvement, and not just rely on media exposure. Schmitt (1999) proposed that customer experiences are conceptualized as a combination of senses, affect, and cognition. The cognitive component comprises the physical attributes or intangible qualities that meet the utilitarian needs, the affective component refers to customers' moods or feelings (e.g., fun or pleasure), and the sensory component can cause excitement and pleasure (Aaker, 1996; Gentile et al., 2007). Experiential marketing researchers have pointed out that companies' long-term competitive advantages can be obtained by continuously exceeding customers' cognitive, emotional, and sensory expectations (Kim and Perdue, 2013; Wiedmann et al., 2018).

The concept of experiential marketing is to ensure consumers' emotional attachment to a brand by engaging their five experiences (namely sense, feel, think, act, and relate; Schmitt, 1999). By using experiential marketing strategies, companies can develop a variety of experiences for their customers. In the sponsorship research, sports sponsorship provides a medium for experiential activation strategies to build long-term emotional connections and bring benefits to brands (Bal et al., 2009; DeGaris et al., 2009). The term “activation” refers to an activity that is used to maximize the effects of sponsorship, and requires immersive participation by the participants (Wohlfeil and Whelan, 2007). The activation of experiential marketing can be achieved through various strategies, such as themed parties, sponsorship-linked roadshows or activities, or experiencing sports by using virtual reality technology (Papadimitriou and Apostolopoulou, 2009; Crowther, 2010). Bjerke and Kirkesaether (2020) proposed a sports sponsorship activation framework and guidelines for sponsors such as sports events and athletes, and proposed the important characteristics and capabilities to attract sponsors. From the perspective of experiential marketing, Schmitt (1999) proposed that sponsoring brand companies need to plan an event environment to enable consumers to participate in brand-related activities through immersive and personally relevant experiences.

### Sponsorship Satisfaction

Satisfaction is generally defined as pleasurable fulfillment (Oliver, 1997). Overall satisfaction is a judgment based on the customer's whole experience during transactions with service providers (Zena and Hadisumarto, 2013). In this sense, satisfaction is a form of the affective and emotional response generated by the consumer's evaluation of consumers, for example, the feeling of liking or disliking a product. However, when consumers try to understand why they like a particular product, customer satisfaction may also consist of cognitive components (Peter and Olson, 1999).

In the service context, satisfaction is greatly affected by the quality of the delivered service, and requires consumers to actively participate in the delivery process (Ueltschy et al., 2007; Deng et al., 2013). In this sense, researchers have suggested that sponsoring companies often have an advantage in terms of engaging event participants and target consumers in brand-related activities (Wohlfeil and Whelan, 2007). In the current research, therefore, we assumed that satisfaction with sports events is largely affected by consumers' perceptions and evaluations of the service quality provided by sponsors during the entire period of participation in sports events.

### Brand Equity

Building a strong brand is the goal of many companies because a large portion of the company's value depends on intangible assets, especially brand equity (Simon and Sullivan, 1993). Aaker (1996) considered brand equity to be comprised of a group of assets related to brands, names, and symbols through which the value of a product or service can be added to a firm's customers. From a marketing perspective, Keller (2001) proposed customer-based brand equity (CBBE) as “the differential effect that brand knowledge has on consumer responses to marketing activity with respect to that brand.” Many measures of brand equity focus mainly on the functional aspects of brand, such as brand awareness, brand performance, and brand judgments. Nam et al. (2011) argued that the existing measurement scales may ignore other essential components of brand equity, and are therefore unsuitable for service-oriented brands. In their research, lifestyle congruence was included in the brand equity dimension related to symbolic consumption. In Keller 2001 study, consumers' feelings (i.e., consumers' emotional reactions to the product) are also considered as one major component of brand equity. In this sense, Alwi and Kitchen (2014) perceived brand as a cluster of rational and emotional values. In the brand management of multisensory marketing, marketing activities that attract the five senses provide opportunities to evoke consumers' favorable emotions, which can be transferred to the brand (Wiedmann et al., 2018).

Scholars in sports brand management have paid increasing attention to brand equity. For example, Ross et al. (2006) asserted that brand awareness is an important concept for understanding brand associations in sports. Bauer et al. (2005) highlighted the importance of brand awareness in team sports, and demonstrated the significant impact of brand equity on the company's economic success. Ross et al. (2006) suggested that brand association and brand awareness contribute to the value of sports brands. Lee et al. (2015) indicated that sponsoring a charitable sporting event can be an effective marketing tool for a company to build brand equity. Although there are many forms of brand equity, from a consumer perspective, Aaker (1996) stated the one-dimensional perceived quality of brand equity. From the perspective of experiential marketing, the current study, like previous studies (Yoo and Donthu, 2001; Ross et al., 2008), also uses a single dimension of brand equity to evaluate the effectiveness of brand performance in marketing events.

### Hypothesis Development

Product or service performance was found to have a direct impact on consumer satisfaction and loyalty (Shaffer and Sherrell, 1997). A positive relationship can also be found between experiential marketing (sense, feel, think, act, and relate) and customer satisfaction (Zena and Hadisumarto, 2013). In this study, the activation of experiential sponsorship enables consumers to receive the sponsor's products (e.g., sportswear, towels, and other outdoor equipment) in advance and experience the quality of the products in a sports competition. Therefore, we assumed that the satisfaction of participants would not only be affected by the service quality of the sports event, but also by the experience of the sponsor's products.

Moreover, research has suggested that an effective event design of an experiential marketing activity can increase consumers' awareness and attitudes as well as brand recall toward the sponsoring brand (Fransen et al., 2013). It was also confirmed by Zarantonello and Schmitt (2013) that consumer experiences of events positively contribute to the sponsoring company's brand equity. Addis and Holbrook (2001) further asserted the significant impact of consumers' perceived experience on their future consumption decisions. By taking into account consumers' experience of sports products or services, Ross et al. (2008) considered the experience-induced consumer experience as an antecedent of brand equity. Sponsorship can act as a catalyst, inducing consumers to have a favorable disposition toward sponsors, and triggering them to actually purchase the sponsor's products (Meenaghan, 2001). The authors thus further pointed out that this product trial opportunity is valuable because it enables consumers to assess the product's merits and thus encourages their future purchase intention. Thus, we formulated the following hypotheses:

H1a: The activation of experiential marketing positively influences participants' satisfaction with sponsorship events.
H1b: The activation of experiential marketing positively influences the brand equity of the sponsoring company.
H1c: The activation of experiential marketing positively influences participants' purchase intention regarding the sponsoring company's products.

Sponsorship is often used as a marketing tool in international or large-scale events to create a positive brand image transferred from the sports events, because effective sponsorship can marginally increase public awareness (Meenaghan, 2013; Nufer, 2016). This is commonly true for well-known companies that have products with high levels of awareness (Crompton, 1996). Zarantonello and Schmitt (2013) confirmed the positive relationship between consumer experience at an event and the sponsoring firm's brand equity. However, when there is low awareness of the brand, we argue that participants' positive attitude toward and satisfaction with the sponsored activity would transfer to the brand and increase their brand awareness of the corporation (Keller, 2001; Sözer and Vardar, 2009).

Many studies have also confirmed the positive relationship between consumer satisfaction and purchase intention (Cronin et al., 2000; Hsiao et al., 2016; Su et al., 2019, 2021). In the sponsorship studies, Wang and Kaplanidou (2013) pointed out that spectators' positive emotions will influence their willingness to purchase the sponsor's products. Previous research pointed out that consumer satisfaction may include both cognitive and affective elements (Peter and Olson, 1999). Sreejesh et al. (2020) further showed that consumers' cognition and feelings generated by events and sponsors can ultimately shape their behavioral responses to sponsors. Specifically, Cheong et al. (2019) confirmed that consumers' attitudes toward sponsorship are an important indicator of their purchase intentions. Inferring from the above research, we assumed that consumers' satisfaction with a small-scale sports sponsorship event would enhance their purchase intention regarding the sponsor's products. Accordingly, we proposed the following hypotheses:

H2a: Satisfaction with sponsorship events positively influences the sponsor's brand equity.
H2b: Satisfaction with sponsorship events positively influences the participants' purchase intention regarding the sponsor's products.

Many researchers have confirmed the essential consequence of brand equity on a firm's performance, such as stock prices (Simon and Sullivan, 1993), market value, and profitability performance (Baldauf et al., 2003). The overall evaluation of brand equity, including cognitive and affective elements, may lead to behavioral intentions and subsequent actual behaviors (Alwi and Kitchen, 2014). Keller (2001) asserted that as brand strength increases, consumers are more likely to purchase branded products and pay a premium price. Referring to sports sponsorship events, prior studies have pointed out that by shifting the image from the sponsored event to the sponsoring company, corporate brands can be enhanced, thereby affecting consumers' willingness to purchase products or services (Crompton, 1994; Chien et al., 2011; Liu et al., 2015). Therefore, we propose the following hypothesis:

H3: The brand equity of sports sponsoring corporations positively influences consumers' purchase intention regarding the sponsoring company's products.

## Methodology

### Participants and Procedure

In this study, a paper-based questionnaire survey method was used to collect real data from actual road race events. Approximately 4,500 people participated in the “Shimen Reservoir International Road Run Challenge” campaign held in Taiwan. All participants were classified into three groups: the carbon reduction half-horse group (21 km), the energy-saving group (11 km), and the perpetual walking group (4 km). This small campaign was organized to call for “Environmental protection, love the earth” and urged people to stay close to the outdoors, save energy, and reduce carbon emissions. This road running event was held by a major outdoor leisure brand in Taiwan, GoHiking.

In the road running events, the sponsors' products (e.g., sportswear and equipment) were used and experienced by participants during the sports competition. Researchers have suggested that the experiential marketing and product performance have a positive impact on participants' satisfaction with the sponsorship events (Zena and Hadisumarto, 2013). Respondents were conveniently selected after participants had completed the race and were in the terminal rest area. Several trained members of staff briefly addressed the academic purpose of this study, obtained permission from the participants, and gave them stationery items worth about 2 US dollars as gifts after completing the survey.

The questionnaire was printed on both sides of an A4 sheet of paper, and the items were divided into two parts. The first was a short greeting, an explanation of the research purpose, the protection of personal information, and then the research items. A total of 308 questionnaires were distributed. As some respondents did not notice the items on the back, we discarded 70 incomplete questionnaires, resulting in an effective questionnaire rate of 77.3%. The composition of male and female participants was 47.9 and 52.1% of the sample, respectively. Most participants were between 21 and 50 years old (73%). About one-third of the respondents participated in the running activity for the first time. More than half of the respondents had not used GoHiking products. Table 1 lists the details of the respondents.

**Table 1:** Characteristics of the sample (*N* = 238 actual participants).

**Characteristics**	**Number**	**Percentage (%)**
**Gender**		
Male	114	47.9
Female	124	52.1
**Age**		
16–20	36	15.1
21–30	55	23.1
31–40	63	26.5
41–50	56	23.5
Over 51	28	11.8
**Number of times participating in running activities in the past**		
0	119	30.7
1	125	32.2
2	76	19.6
Over 3	68	17.5
**Have used GoHiking products**		
Yes	107	45.0
No	131	55.0

### Measurements of the Constructs

Measurement items in this study were modified from previous studies using the Likert scale with anchors ranging from 1 (*strongly disagree*) to 7 (*strongly agree*). Ten items of brand experience related to the user experience of GoHiking products (e.g., running clothes and backpacks) were drawn from Brakus et al. (2009). Three items of satisfaction with the sponsored race were modified from Mimouni-Chaabane and Volle (2010). Three items of brand equity were adopted from Lee et al. (2015). Three items of purchase intention were drawn from Venkatesh et al. (2003). We adopted the backward translation technique (converting the original items from English to Chinese, and then back to English) to resolve any inconsistencies between the two versions. A pretest was performed involving 40 students. We used the results of the pretest to make minor text corrections, including removing one item from experiential marketing due to low standardized loading. The final version is provided in Appendix A.

## Empirical Results

### Results of the Measurement Model

In this study, we adopted confirmatory factor analysis and additional tests to assess the reliability and validity of the measures (e.g., composite reliability, convergent validity, and discriminant validity). We used the statistical software SmartPLS (version 3.32) to implement the data analysis in this study (Ringle et al., 2015). In terms of scale reliability, the composite reliability (CR) with confirmatory factor analysis was employed. The CR measures how consistently individuals respond to the measurement items within a construct. The results showed that the values of CR (ranging from 0.92 to 0.94, as shown in Table 2) were all above the threshold of 0.7 (Hair et al., 1998), indicating high reliability of the measurement.

**Table 2:** The composite reliability.

**Indicators**	**Standardized loading**	***t* value**	**CR**	**AVE**
EM_1_	0.79	27.25	0.94	0.64
EM_2_	0.83	33.65		
EM_3_	0.85	34.13		
EM_4_	0.84	35.50		
EM_5_	0.82	26.93		
EM_6_	0.80	23.69		
EM_7_	0.81	23.69		
EM_8_	0.74	19.54		
EM_9_	0.74	22.42		
EM_10_	0.81	27.06		
SA_1_	0.82	15.08	0.94	0.84
SA_2_	0.85	15.93		
SA_3_	0.94	18.77		
BE_1_	0.82	15.08	0.94	0.83
BE_2_	0.85	15.93		
BE_3_	0.94	18.77		
PI_1_	0.81	14.56	0.92	0.79
PI_2_	0.83	15.27		
PI_3_	0.84	15.37		

*EM, experiential marketing activation; SA, satisfaction with the sponsorship event; BE, brand equity of the sponsoring company; PI, purchase intention regarding the sponsor's products*.

Convergent validity was obtained as all factor loadings of items within a construct were statistically significant (the t-values ranging from 15.08 to 35.50). It was also assured with all values of the average variance extracted (AVE) exceeded 0.5 (Hair et al., 1998). On the other hand, the discriminant validity is achieved if the square root of the AVE for each construct is larger than its correlation coefficients for all other constructs (Fornell and Larcker, 1981). As shown in Table 3, the square roots of AVE for all constructs, ranging from 0.80 to 0.91, were larger than other correlations. The results suggest that the instrument had proper convergent and discriminant validity.

**Table 3:** Descriptive statistics, variance explained, and correlations.

	**Means**	**Standard deviation**	**EM**	**SA**	**BE**	**PI**
EM	5.30	0.89	**0.80**	-	-	-
SA	5.66	1.04	0.60^***^	**0.92**	-	-
BE	5.38	0.93	0.68^***^	0.57^***^	**0.91**	-
PI	5.11	0.95	0.66^***^	0.45^***^	0.60^***^	**0.89**

*** *p < 0.001; On-diagonals are square roots of AVE (boldface)*.

*EM, experiential marketing activation; SA, satisfaction with the sponsorship event; BE, brand equity of the sponsoring company; PI, purchase intention regarding the sponsor's products*.

Last, the potential common method bias was examined by utilizing Harmon's single factor test (Harman, 1967; Podsakoff and Organ, 1986). The test of factor analysis shows that no single factor occurs because the first factor had explained 51.6% of the total variance, which was over the threshold value of 50%. In addition, a potential common method bias was also assessed by a full collinearity test (Kock and Lynn, 2012). The variance inflation factors (VIFs) for all of the constructs (between 1.98 and 3.89) are below the recommended threshold of 5 (Hair et al., 2011). This suggests that common method variance was less of a concern.

### Results of the Structural Model

With a proper measurement model, the hypothesized relationships of the structural model (see Figure 1) were examined using SmartPLS with the bootstrapping approach (5,000 samples with replacement). Table 4 lists the results of the structural model, including path coefficients, standard errors, and the upper and lower bounds of the 95% confidence interval. In this study, SmartPLS with the bootstrapping approach was also used to estimate the direct effects, indirect effects, and total effects between experiential marketing activation, satisfaction with the sponsorship campaign, brand equity, and purchase intention. If the result of estimated confidence intervals contains zero, the path coefficient was suggested to have no difference from zero and thus the two constructs are considered to not have a significant relationship in the model.

**Table 4:** Hypothesis testing results and the direct, indirect, and total effects in the model.

**Hypothesis and effect**	**Path**			**95% confidence interval**	
		**Bootstrap estimate**		**Bootstrap percentile**	
		**β**	**S.E**.	**Lower**	**Upper**
H1a direct effect	EM → SA	0.60^***^	0.05	0.50	0.70
H1b direct effect	EM → BE	0.53^***^	0.06	0.40	0.65
H1c direct effect	EM → PI	0.48^***^	0.07	0.34	0.62
H2a direct effect	SA → BE	0.25^***^	0.06	0.12	0.37
H2b direct effect	SA → PI	0.002	0.06	−0.12	0.12
H3 direct effect	BE → PI	0.28^***^	0.07	0.13	0.42
Indirect (mediated) effect	EM → SA → BE	0.15^***^	0.04	0.07	0.23
Indirect (mediated) effect	EM → BE → PI	0.14^***^	0.04	0.06	0.23
Indirect (mediated) effect	SA → BE → PI	0.07^*^	0.03	0.02	0.12
Indirect (mediated) effect	EM → SA → BE → PI	0.04^*^	0.02	0.01	0.07
Indirect (mediated) effect	EM → SA → PI	0.001	0.04	−0.07	0.07
Total effect (EM → PI)^a^		0.66^***^	0.05	0.57	0.76
Total effect (SA → PI)^b^		0.07	0.06	−0.05	0.20

* *p < 0.05*;

** *p < 0.01*;

*** *p < 0.001*.

*BE, brand equity of the sponsoring company; PI, purchase intention regarding the sponsor's products; EM, experiential marketing activation; SA, satisfaction with the sponsorship event*.

a *Total effect (EM → PI): (EM → PI) + (EM → BE → PI) + (EM → SA → PI) + (EM → SA → BE → PI)*.

b *Total effect (SA → PI): (SA → PI) + (SA → BE → PI)*.

As shown in Table 4, the path results show that five out of the six hypotheses are significant, supporting the direct effects from empirical marketing to satisfaction, brand equity, and purchase intention in the context of small sports sponsorship. First, empirical marketing activation has a positive and significant impact on the satisfaction of sponsored activities (β = 0.60, *p* < 0.001, H1a was supported), brand equity (β = 0.53, *p* < 0.001, H1b was supported), and purchase intention regarding the sponsor's products (β = 0.48, *p* < 0.001, H1c was supported). Next, participants' satisfaction with the sports campaign also had a positive impact on brand equity (β = 0.25, *p* < 0.001, H2a was supported); however, it failed to influence their purchase intention regarding the products of the sponsoring company (β = 0.002, *p* > 0.05, H2b was not supported). Finally, it is also confirmed that brand equity had a positive impact on the purchase intention regarding the products of the sponsoring company for the small event (β = 0.28, *p* < 0.001, H3 was supported).

In this study, a mediation analysis was also performed to test the potential indirect effects among the research constructs. In line with previous sports management literature (Hedlund, 2014), a structural equation modeling (SEM) with the bootstrapping procedure was used. The advantage of using SEM with the bootstrapping method is that it can provide direct and indirect effects simultaneously, thereby strengthening the results of the structural model and decomposing the construct relationships of small sponsorship in this present study. The results showed that four out of five mediated paths were positive and significant in the proposed model, including: (1) experiential marketing, satisfaction, and brand equity (β = 0.15, *p* < 0.001), (2) experiential marketing, brand equity, and purchase intention (β = 0.14, *p* < 0.001), and (3) satisfaction, brand equity, and purchase intention (β = 0.07, *p* < 0.05). It is also noteworthy that the fourth significant indirect effect is a long path between experiential marketing and purchase intention through satisfaction and brand equity (β = 0.04, *p* < 0.05). However, the indirect path between experiential marketing, satisfaction, and purchase intention was not significant (β = 0.001, *p* > 0.05).

Based on the estimated coefficients of direct and indirect paths, the total effects were calculated. The total effects (from experiential marketing to purchase intention) include four path effects: the direct relationship (experiential marketing → purchase intention) and indirect relationships (experiential marketing → brand equity → purchase intention; experiential marketing → satisfaction → purchase intention; experiential marketing → satisfaction → brand equity → purchase intention) were positive and significant (β = 0.66, *p* < 0.001). However, the total effects for the direct relationship (satisfaction → purchase intention) and the indirect relationship (satisfaction → brand equity → purchase intention) were not supported (β = 0.07, *p* > 0.05).

Finally, based on the results of variance explained (*R*^2^), the explanatory power of three endogenous constructs in the proposed model was evaluated. First, the results showed that 36% of the variance in satisfaction with the event was accounted for by experiential marketing activation. In addition, 50% of the variance explained in brand equity was accounted for by both experiential marketing activation and satisfaction with the event. Finally, 48% of the variance explained in purchase intention was accounted for by experiential marketing activation, satisfaction, and brand equity. More detailed information about the empirical results of the structural model is summarized in Table 4.

## Discussion

The main purpose of this study is to examine the effectiveness of small sports event sponsorship from the perspectives of experiential marketing activation, sponsorship satisfaction, brand equity, and purchase intention. Based on the analysis of the actual participants' responses, a proposed research model was presented and it was found that the experiential marketing mechanism to engage consumers in product experiences, the satisfaction of participants with sponsored activities, brand equity, and subsequent purchase intentions. In addition, six research hypotheses among four constructs were examined using SEM with bootstrapping procedures. The results showed that five of them were supported. Based on the results, we drew the following conclusions for this study: the activation of experiential marketing played a decisive role in the participants' satisfaction with the event, the brand equity of the sponsor, and the purchase intention regarding the sponsor's products. Satisfaction had a direct impact on brand equity as well; however, only indirect effect through brand equity on purchase intention was found. Overall, the proposed model has a relatively high explanatory power for sponsorship satisfaction (36%), brand equity (50%), and purchase intention (48%).

This study contributes to the literature in three ways. First, by focusing on small sports sponsorships, this study confirmed the effectiveness of sponsorship activities for brand equity and subsequent product purchase intentions. The research results can provide supplementary evidence for the sports sponsorship literature obtained in mega-scale sports event research. Second, the current research objects were actual participants in sports competitions, rather than spectators or ordinary consumers. Therefore, the research results have a more realistic basis. Hickman (2020) pointed out that the brand awareness displayed by the participants of the sponsor brand experience is higher than that of television audiences. Third, in line with previous study, researcher has suggested that the results of consumer-oriented sponsorship marketing events as cognition, affection, and behavior (Cornwell, 2019). As far as we are aware, this is a pioneering study that combines the above attributes in terms of the activation of experiential marketing, satisfaction with the sponsorship events, and brand equity theories to explore the effectiveness of sports sponsorship. Fourth, the mediation test shows that brand equity is an important mediator of experience marketing and satisfaction to product purchase intention.

The following discussions are provided. First, regarding the main interest of experiential marketing activation in the context of small sports sponsorship, we found that the activation of experiential marketing plays the most important role in affecting other variables, including satisfaction with sponsored activities, brand equity, and purchase intention. This finding is consistent with previous studies (Shaffer and Sherrell, 1997; Zena and Hadisumarto, 2013) which found that experiential marketing and product performance have a positive impact on participants' satisfaction with the sponsorship events. Meenaghan and O'Sullivan (2013) emphasized the importance of brand experience, engagement, and involvement in the evaluation of sponsors. Moreover, like many other research findings, the activation of experiential marketing has a strong impact on brand equity and the purchase intention of sponsoring brands (Ross et al., 2008; Fransen et al., 2013). Based on the results, the current study suggests that companies can design some experiential marketing activities related to the sponsored events to enhance consumers' engagement in the product experiences, and can thereby induce their emotional attachment to the sponsor's brand. As such, positive outcomes will be generated through brand equity and purchase of sponsored products.

Next, participants' satisfaction with the event had a positive impact on the sponsor's brand equity. This result is consistent with previous studies on international or large-scale sponsorship events (Sözer and Vardar, 2009; Zarantonello and Schmitt, 2013). Based on this finding, we encourage companies with limited resources and low brand awareness to organize sponsorship events to increase potential users' participation and experience. A well-designed sports event will make participants feel satisfied with the event and might transfer this positive emotion to the sponsored brand. However, it should be noted that, unlike our expectations and previous studies (Wang and Kaplanidou, 2013; Cheong et al., 2019; Sreejesh et al., 2020), participants' satisfaction with the event in the proposed model did not significantly affect their purchase intentions. We speculate that there are two reasons. These studies mainly focused on globally or nationally well-known brands and large-scale sports events, and the research objects were audience members or ordinary consumers. In the current study, more than 55% of subjects had never used the sponsor's products. Therefore, in order to convert participants' satisfaction with the event into their willingness to buy the sponsor's products, there should be an intermediate variable, namely brand equity.

Third, our results showed that brand equity has a significant impact on purchase intentions. Previous researchers have suggested that consumers are more likely to buy branded products as the company's brand equity increases (Baldauf et al., 2003; Alwi and Kitchen, 2014). In this study, the results revealed that sponsoring sports events is an effective way to increase sponsors' brand equity, thereby influencing consumers' intention to purchase the sponsor's products, even making them willing to pay for some other branded products (Keller, 2001). This study also performed some in-depth analysis of the direct and indirect effects between constructs. The results showed that the direct effects from experiential marketing activation to brand equity and from experiential marketing activation to purchase intention were empirically larger than the same indirect paths mediated by satisfaction and brand equity. This finding provides some empirical evidence for small-scale companies considering the marketing strategy of incorporating experiential marketing into sponsoring sports events, which will directly increase the company's brand equity and willingness to consume brand products. In addition, the direct path from satisfaction to purchase intention is fully mediated by brand equity. In other words, participants' awareness and knowledge of the sponsored brand (compared to those who do not know the sponsored brand) has a positive impact on their intention to purchase the company's products. In conclusion, companies should consider sponsoring or holding frequent small-scale sports events to encourage more people to participate in the sponsored activities, thereby establishing a deeper connection between sponsors and events, and influencing the purchase decisions of potential consumers.

## Practical Implications

The literature has highlighted the importance of the perspective of actual participants (Hickman, 2020). In this study, our findings aim to provide some empirical evidence for the sponsorship literature on how small sponsorship events can benefit sponsoring companies both intangibly (e.g., brand equity) and tangibly (e.g., product purchase). At the same time, it brings affective and cognitive benefits (e.g., brand product experience, satisfaction, and brand equity) to the company.

In this study, we found that experiential marketing activation can enhance the sponsor's brand equity and increase participants' subsequent purchase intentions. Therefore, the company can enhance participants' product experience before and during sports events. For example, the sports package (including sportswear, towels or small backpacks) sent to contestants before the competition provides an excellent opportunity for them to experience and evaluate the company's products. For participants or escorts, due to their satisfaction with the event, this is also a good opportunity to increase their good impression of the sponsoring company. According to Meenaghan (2001), a successful sports sponsorship event can provide consumers with a good impression of sponsors, thereby increasing the awareness and recognition of sponsored brands. Therefore, a small sponsorship event is a valuable practice for small-sized companies or newly established brands, such as small-scale amateur (Low and Pyun, 2016) and small-scale marathon events (Koo et al., 2014). Such events can also encourage community residents who are amateur players to participate in the activity, which will provide more exposure opportunities for sponsoring brands.

## Limitations and Future Study

The study has some limitations which provide directions for future research. First, the data were collected from only one event held by a major outdoor leisure company, GoHiking, in Taiwan, and it was the first time for GoHiking to sponsor this event. The visibility of sponsors or sponsored events is limited, which in turn limits the extent to which the results can be generalized. Therefore, future research can consider sporting events with years of sponsorship to test the impact of sponsorships on brand equity and purchase intention. In addition, the study was carried out on a convenience sample. Even though the participants were fairly well-distributed across the sample attributes such as gender and age, nearly one-third of the respondents were participating in this kind of activity for the first time. Future research can consider distinguishing participants into recreational and repeat event runners for individual or comparative research.

Second, we conducted this study in Taiwan. Many large-scale sporting events (e.g., international marathons or triathlons) often attract sports fans from different countries. Therefore, it is suggested that this study be replicated in different cultural contexts to test the generalizability of the findings. Moreover, the scale was developed in the Chinese language, so further validity and reliability testing should be done. Split-half testing can be used to measure reliability. However, to divide the research data into two parts, the sampling size must be at least 400. Therefore, it is expected that more data will be collected by distributing questionnaires in different geographic regions for future research purposes. Nevertheless, the current research uses composite reliability, convergent validity, and discriminant validity to evaluate measurement models like most research.

## Data Availability Statement

The original contributions presented in the study are included in the article/supplementary material, further inquiries can be directed to the corresponding author.

## Ethics Statement

Ethical review and approval was not required for the study on human participants in accordance with the local legislation and institutional requirements. Written informed consent for participation was not required for this study in accordance with the national legislation and the institutional requirements.

## Author Contributions

All authors contributed to the conception of the idea, implementing and analyzing the experimental results, writing the manuscript, and read and approved the final manuscript.

## Conflict of Interest

The authors declare that the research was conducted in the absence of any commercial or financial relationships that could be construed as a potential conflict of interest.

## Appendix

**Appendix A:** Measure of constructs.

**Construct**
**Activation of experiential marketing (EM)**
After using the products of GoHiking…
EM1. I find this brand interesting from a sensitive point of view.
EM2. I find this brand attractive.
EM3. This brand makes me experience sensations and feelings.
EM4. When I visualize this product, I would like to take actions (e.g., purchase).
EM5. This brand provides physical experiences.
EM6. This brand is action-oriented.
EM7. I get plenty of ideas when I find this brand.
EM8. This brand makes me think.
EM9. This brand stimulates my curiosity and problem solving.
EM10. This is an emotional brand.
**Sponsorship satisfaction (SA)**
SA1. I made a good choice when I decided to participate in this running race.
SA2. The advantages I receive from being a participant of this program meet my expectations.
SA3. All in all, I am satisfied with this running race.
**Brand equity of the sponsoring company (BE)**
BE1. After using this brand, I am very likely to grow fond of it.
BE2. I have positive personal feelings about this brand.
BE3. With time, I will develop warm feelings toward this brand.
**Purchase intention regarding the sponsoring products (PI)**
PI1. I intend to use GoHiking's products in the future.
PI2. I will always try to buy GoHiking's products in my daily life.
PI3. I plan to buy GoHiking's products frequently.
